# The distance of the gluteal nerve in relation to anatomical landmarks: an anatomic study

**DOI:** 10.1007/s00402-017-2847-z

**Published:** 2017-11-25

**Authors:** David Putzer, Matthias Haselbacher, Romed Hörmann, Martin Thaler, Michael Nogler

**Affiliations:** 1 Experimental Orthopaedics, Department of Orthopaedic Surgery, Medical University of Innsbruck, Innrain 36 15, 6020 Innsbruck, Austria; 2 Department of Trauma Surgery, Medical University of Innsbruck, Anichstrasse 35, 6020 Innsbruck, Austria; 3 Division clinical and functional anatomy, Department of Anatomy Histology and Embryology, Medical University of Innsbruck, Anichstrasse 35, 6020 Innsbruck, Austria; 4 Department of Orthopaedic Surgery, Medical University of Innsbruck, Anichstrasse 35, 6020 Innsbruck, Austria

**Keywords:** Gluteal nerve, Direct anterior approach, Minimaly invasive hip arthoplasty, Avoiding nerve lesions, Gluteal insufficiency

## Abstract

**Introduction:**

Gluteal insufficiency is of concern with lateral approaches to total hip arthroplasty. Damage to the branches of the superior gluteal nerve may cause degeneration of the innervated muscles. The direct anterior approach exploits the intermuscular and internerval interval between tensor fasciae latae laterally and sartorius and rectus femoris muscle medially. In this study, the distance of the superior gluteal nerve in relation to anatomical landmarks was determined.

**Materials and methods:**

Two experienced surgeons implanted trial components in 15 alcohol glycerol fixed cadavers with 30 hips. The trials were removed, and the main branch of the superior gluteal nerve and muscular branches of the nerve were exposed from lateral.

**Results:**

No visual damage to the main nerve branches and the location of the nerve in relation to the greater trochanter were noted by an experienced surgeon. The superior gluteal nerve and its muscular branches crossed the muscular interval between the gluteus medius and tensor fasciae latae muscles at a mean distance of 39 mm from the tip of the greater trochanter.

**Conclusions:**

The direct anterior approach for total hip arthroplasty minimizes the risk of injuring the superior gluteal nerve, which may result in a gluteal insufficiency. Special care should be paid on avoiding overstretching the tensor fasciae latea muscle using minimum force on retractors during surgery and by taking care of the entrance point of the superior gluteal nerve to the tensor fasciae latae.

## Introduction

One of the major drawbacks of the lateral approaches to the hip introduced over the last decades [1–3] is that they affect the hip abductor muscles negatively. They come close to the branches of the superior gluteal nerve [4–6] where lesions can result in partial or total fatty degeneration of the gluteus medius, minimus and tensor fasciae latae.

Pfirrmann et al. reported a total fatty generation in result of a direct muscle damage or to failure to restore the gluteal insertion after a transgluteal approach [7]. Muscle atrophy is an important differential diagnosis relative to simple tendon tear in patients with limping, which may be caused also by damage to the superior gluteal nerve after lateral approach hip surgery [7]. In a prospective study of Ramesh et al. involving 81 consecutive patients who underwent lateral approach total hip arthroplasty, the abductor muscles of the hip were assessed electrophysiologically and clinically. Results showed that in nine patients, complete denervation occurred [4]. Barrack et al. states that sciatic nerve injury is the most common nerve injury following THA. Femoral nerve injury is mainly associated with an anterior approach, with a generally better prognosis than with sciatic nerve injury, while the superior gluteal nerve is at risk during the direct lateral approach [8].

Because the gluteus medius and minimus are the main abductors of the hip joint insufficiencies can lead to a decrease in pelvic stability. In severe cases, this can cause clinically relevant weakness and a positive Trendelenburg sign, the inability to stabilize the pelvis in the stance phase of the gait cycle. The resultant limping greatly reduces patient quality of life and is considered a major complication in total hip arthroplasty [9].

Therefore, attempts have been made to perform less-invasive total hip arthroplasty using muscle-preserving approaches such as the direct anterior approach [10–12]. In comparison to the Watson–Jones or anterolateral interval [13–17] the Smith–Peterson interval or direct anterior approach is an intramuscular and internerval approach [18–23]. Although both intervals are muscle-preserving methods [9], only the direct anterior approach is truly internerval. The muscle medial, sartorius and rectus femoris, are innervated by branches of the femoral nerve, those lateral, tensor fasciae latae, gluteus medius and minimus are innervated by the superior gluteal nerve. In the anterior approach, access to the hip joint is obtained by passing between the sartorius (femoral nerve) and tensor fasciae latae (superior gluteal nerve) [24]. The gluteal nerve is the only motoric nerve that exits superior to piriformis muscle and then divides into a superior and an inferior branch [25]. It derives from the posterior braches of the ventral rami of the fourth and fifth lumbar and the first sacral spinal nerves. The gluteal nerve is supplying the gluteus medius, gluteus minimus, and tensor fasciae latae muscles. The gluteus medius and minimus muscle are both innervated by the superior and inferior braches. The terminal branches of the inferior branch are innervating tensor fasciae latea which runs anteriorly to it [26].

Retraction of soft tissue can exert considerable forces on the surrounding tissues especially in minimally invasive approaches, where only a small incision is given [27]. Retraction can cause significant damage to the tissue and result in postoperative acute or chronic pain—due to compression neuropraxia [28–30]. It is highly recommended to use special care with surgical instruments, especially retractors, to avoid excessive retraction. Excessive retraction can be exerted primarily during dislocation of the hip. Supporting the limb throughout the operation is highly recommended [31].

Our aim was to localize the superior gluteal nerve in relation to anatomical landmarks as the anterior superior iliac spine (ASIS), the greater trochanter (GT) and the iliac tubercle (IT). The gluteal nerve was exposed after instrumentation of hip arthroplasty via the direct anterior approach to visualize, if the nerve was injured by the direct anterior approach [20, 23, 32].

## Materials and methods

### Surgical technique

**Fig. 1:**
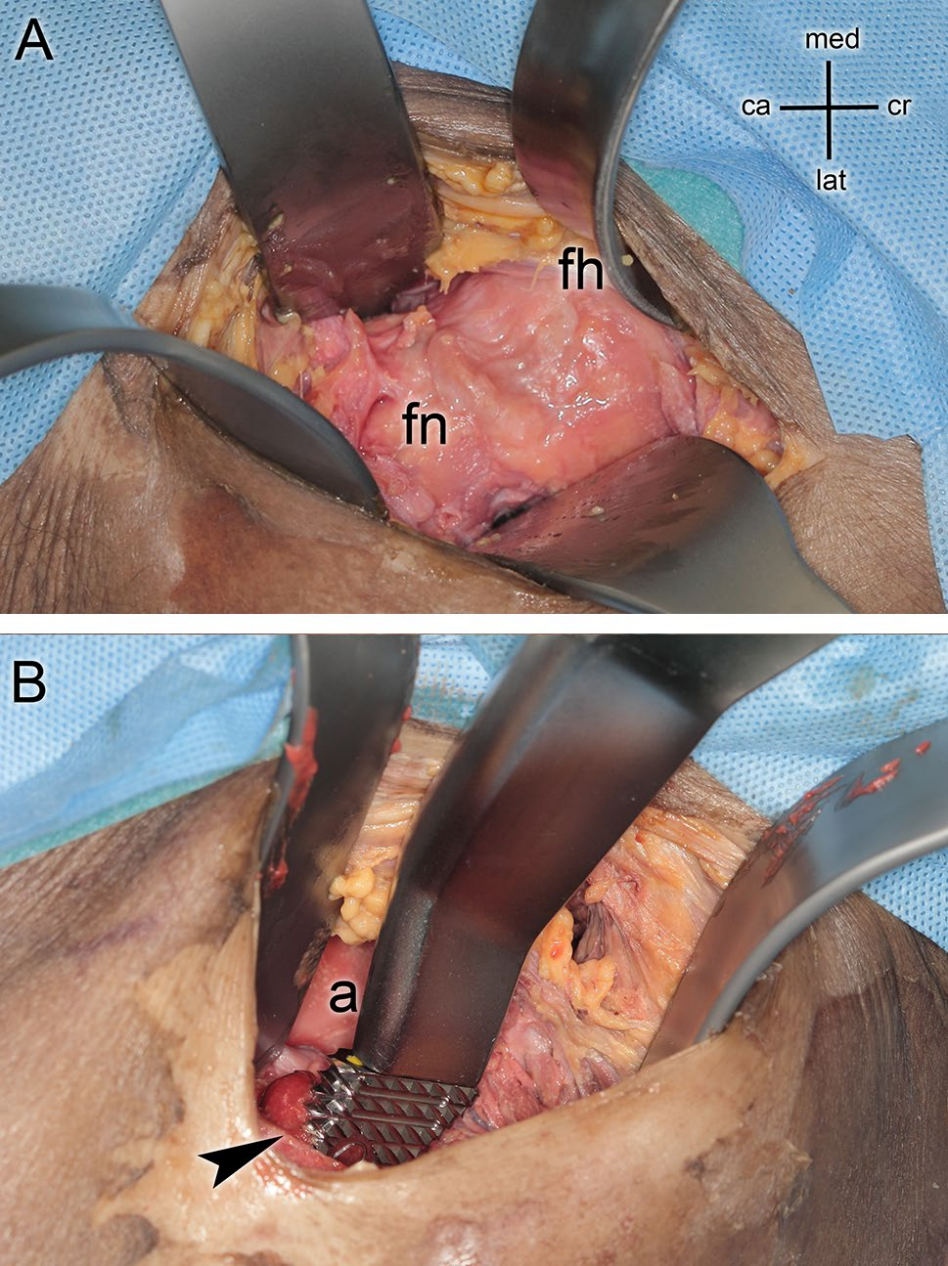
**a** The view during hip arthroplasty using a direct anterior approach on the femoral head (fh) and the femoral neck (fn). Four retractors are placed around the acetabular rim. **b** The preparation of the femoral canal (arrows indicates resection level of the femoral neck) with a broach mounted on a double offset broach handle. Two retractors are placed medial and lateral of the neck. The reamed acetabulum **a** is visible between the surgical instruments

Two experienced surgeons (performing the DAA approach since 2002 and approximately 120 cases per year) who perform the procedure regularly operated on 15 full body alcohol-fixed human cadavers (eight men, seven women). The specimens were placed supine on a standard operating table. No traction tables were used as described by Judet and Judet [33]. All steps were carried out according to the Direct Anterior Approach technique described by Nogler et al. [10] The starting point of the skin incision was 3 cm lateral and 2 cm distal to the ASIS. Subcutaneous fat tissue and the fascia of the tensor fasciae latae were carefully split. The anterior flap of the fascia of the tensor muscle was lifted up and bluntly separated from the muscle. All further preparation was performed subfascially. Four retractors were positioned to expose the capsule: one medial to the tensor muscle and lateral to the capsule, one pointed towards the greater trochanter area, the third medial to the neck, and the fourth retractor under the rectus muscle against the anterior acetabular rim (Fig. 1a).

After excision of the anterior capsule, a neck osteotomy was performed, the head was removed and the acetabulum was reamed to the correct size with hemispherical reamers. A Trident Hemispherical cup© (Stryker, Mahwah, NJ, USA) was implanted. Instruments with offset were used for reaming and impaction. The dorsolateral portion of the capsule was then removed and a double-pronged femoral elevator was positioned dorsal to the greater trochanter to expose the femoral canal. To support the exposure of the femoral canal, the leg was 30°–40° hyperextended, adducted and externally rotated. The preparation of the femoral cavity was performed with a double offset broach handle and broaches of the appropriate size (Fig. 1b) [22, 34]. An Accolade TMZF© stem (Stryker, Mahwah, NJ, USA) of the appropriate size was implanted.

### Anatomical preparation and evaluation

**Fig. 2:**
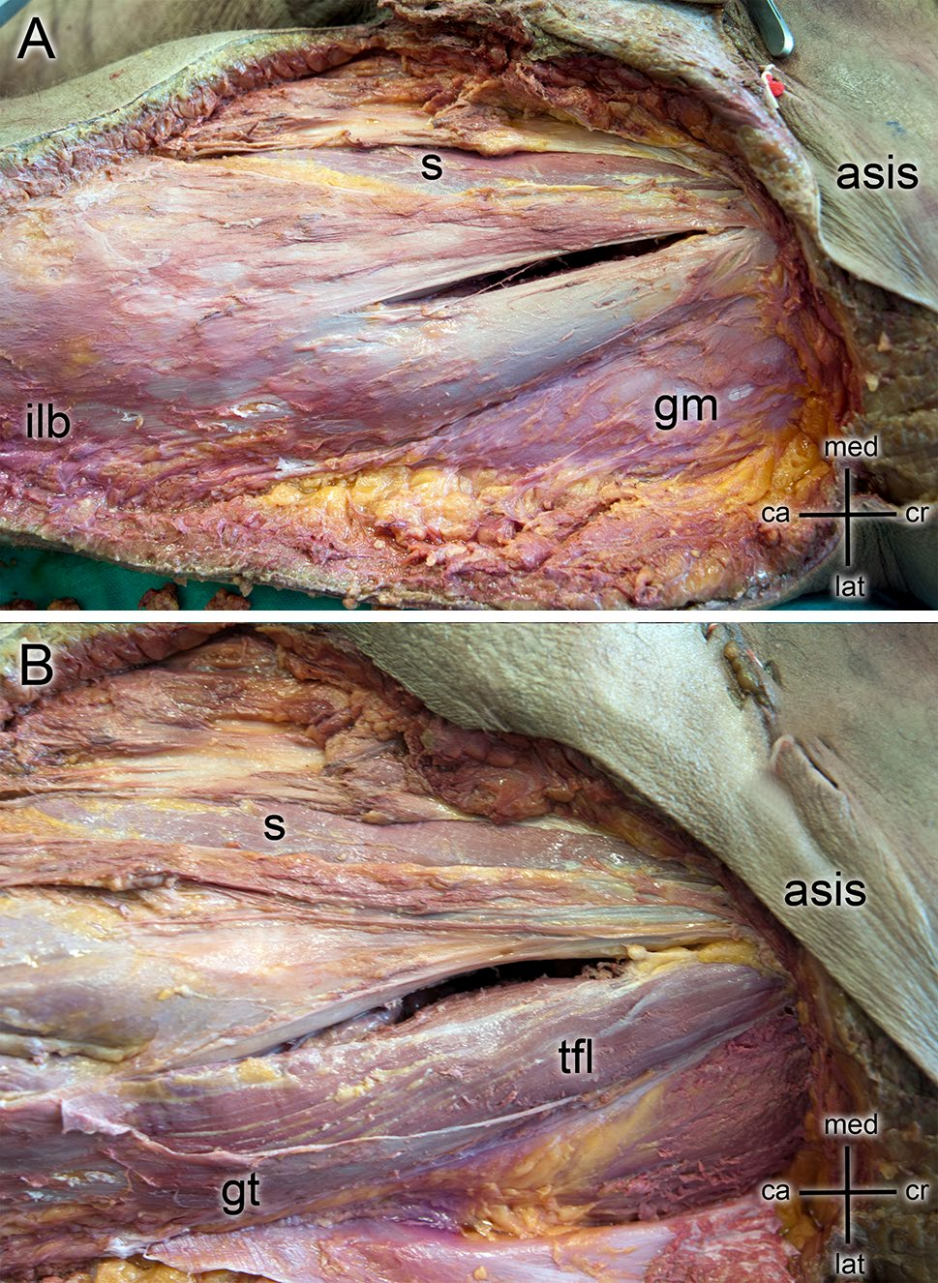
**a** The dissection of a left hip with a direct anterior approach performed. Incision was performed 3 cm lateral and 2 cm distal in reference to the anterior iliac superior spine (ASIS). Sartorius (s), gluteus medius (gm) and the iliolingiunal band (ilb) were exposed. **b** Tensor fascie latae (tfl) was exposed and used as a reference to indicate nervus gluteus superior (NGS) as well as the greater trochanter (gt) and gluteus medius (gm)

**Fig. 3:**
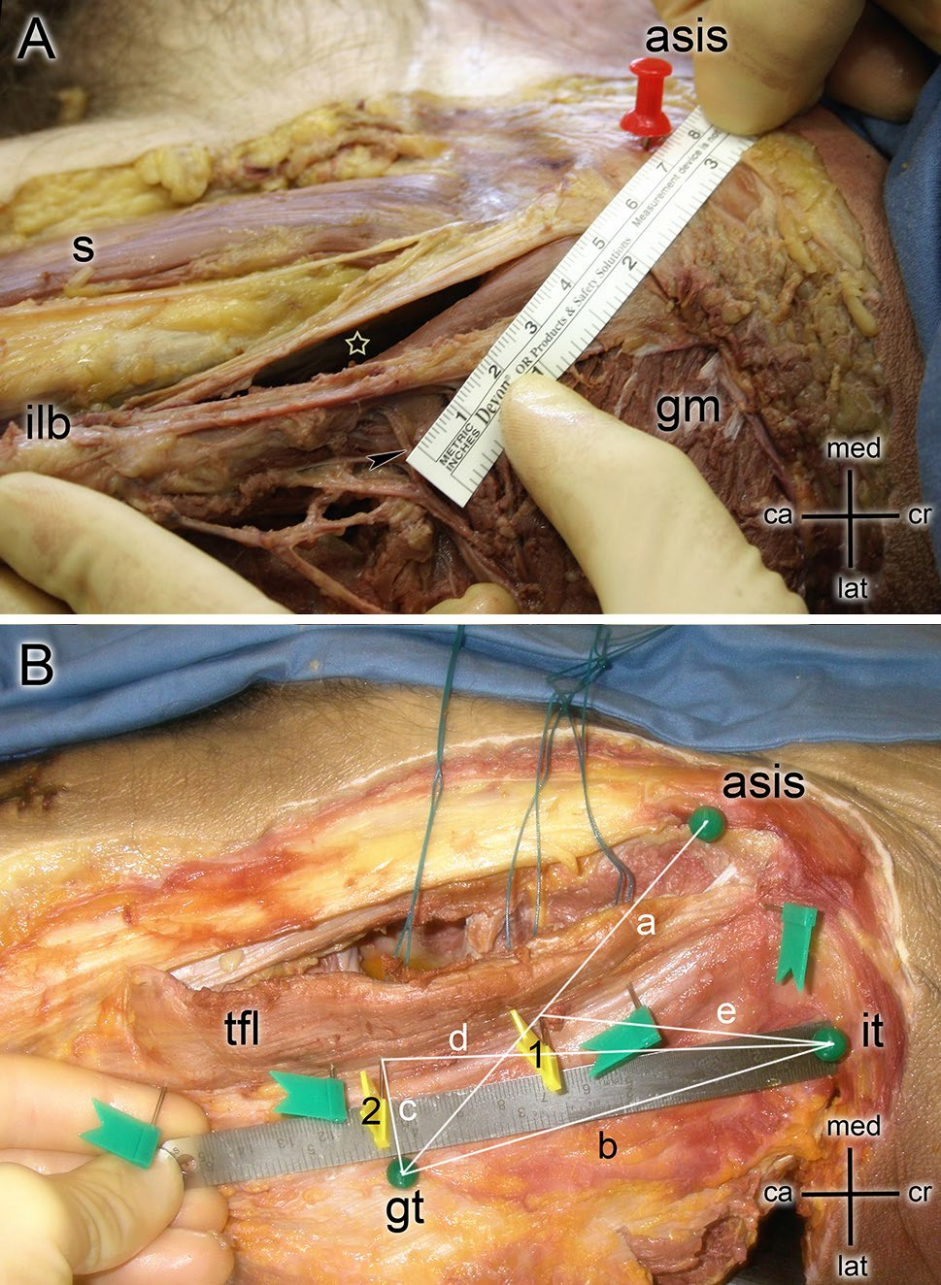
**a** The dissection of a left hip indicating a direct anterior approach (white star) in reference to sartorius (s), iliolingiunal band (ilb) and gluteus medius (gm) muscles. A measurement between ASIS and the insertion point of nervus gluteus superior (black arrow) is shown. **b** Green Balls are indicating the anterior superior iliac spine (asis), the iliac tubercle (it) and the greater trochanter (gt). Green flags show the posterior border of the tensor fascia latae muscle (tfl). The two yellow flags indicate the insertion points of the nervus gluteus superior. (1) indicates the proximal (PSGN) and (2) the distal superior gluteal nerve (DSGN) crossing the TFL. Lines show the measurements indicating (a) Distance between ASIS and GT, (b) distance between GT and IT. (c) distance between the main branch of the superior gluteal nerve (DSGN) and the GT, (d) distance between the main branch of the superior gluteal nerve (DSGN) and IT and (e) distance between the muscular branches of the superior gluteal nerve (PSGN) and IT

The skin was removed after surgery and the tensor fasciae latae, rectus femoris, gluteus medius and gluteus maximus were exposed (Fig. 2a). The gluteus medius muscle was detached from the greater trochanter area and the main branches of the superior gluteal nerve exposed and were inspected for lesions. The ASIS, iliac tubercle (IT), tip of the greater trochanter (GT), and posterior border of the tensor muscle were marked with pins and considered as reference points (Fig. 3a). The following distances were measured (Fig. 3b):

- Distance between ASIS and GT
- Distance between GT and IT.
- Distance between the main branch of the superior gluteal nerve (DSGN) and the GT
- Distance between the main branch of the superior gluteal nerve (DSGN) and IT
- Distance between the muscular branches of the superior gluteal nerve (PSGN) and IT

Mean, range and standard deviation (SD) of the distances were calculated. The soft tissue next to the insertion points of the retractors were carefully examined by a third-experienced surgeon, who was not involved in the surgical procedure, for any visible damage.

Range and mean of body mass index (BMI) and the mean of the age over all specimens were calculated. Descriptive statistics were calculated using SPSS (Version 17.0.1 IBM, USA).

## Results

The sample consisted of 15 alcohol-fixed human cadavers (eight men, seven women) with a mean age of 69 years and a mean BMI of 29 (18–40).

The superior gluteal nerve and its muscular branches were found and exposed in all cases. The branches crossed the muscular interval between the gluteus medius and tensor fasciae latae muscles at a mean distance of 39 mm from the tip of the greater trochanter. The distances between the superior gluteal nerve and the greater trochanter, the iliac tubercle and the anterior superior spine are shown in Table 1. No macroscopically visible damage or transection of the branches of the superior gluteal nerve could be detected.

**Table 1:** The distances in mm between the anatomical reference points (ASIS, GT and IT) are reported as mean ± standard deviation and (range)

Measurement parameter	Distance (mm)
(a) Distance between ASIS and GT	116 ± 8 (18–40)
(b) Distance between GT and IT	113 ± 13 (85–145)
(c) Distance between distal superior gluteal nerve and GT	39 ± 14 (19–61)
(d) Distance between distal superior gluteal nerve and IT	101 ± 25 (40–160)
(e) Distance between proximal superior gluteal nerve and IT	69 ± 25 (20–120)

## Discussion

In terms of patient satisfaction and outcome assessments, total hip replacement is one of the most successful surgeries. However, problems remain such as leg length discrepancy, recurrent dislocation and early loosening. Should gluteal insufficiency develop it can be a severe problem for the patient. Damage to the abductor muscles either directly due to the surgical approach or as a consequence of superior gluteal nerve lesions can be clinically relevant. Avoidance of such damage must be the fundamental goal of any minimally invasive surgical procedure and the patients must be assessed for negative effects on muscle tissue postoperatively. Clinical functional outcome tests have to be performed to demonstrate that the minimally invasive approach has achieved this objective [35].

Ince et al. reported the gluteal superior nerve, leading to the gluteal minimus muscle was 33 (20–50) mm from the tip of the greater trochanter [36]. The nearest point of the superior gluteal nerve branches from the tip of the greater trochanter was on average 19 mm, while a distal branch was found, which was up to 60 (maximum) mm away from the tip of the greater trochanter [36]. Other studies report distances from the greater trochanter to the inferior branch of the superior gluteal nerve ranged from 20 to 30 mm [37] up 60–80 mm [38]. These findings match well with our measurements of the nerve length being on average 39 mm (19–61). The incision starting point was 3 cm lateral and 2 cm distal to the ASIS and was orientate along the longitudinal axis of the TFL muscle. The gluteal superior nerve and its branches are not interfering with the interval. Ince et al. also reported that using the Watson Jones interval runs the risk of damaging the branch of the gluteal superior nerve and that a safe zone is hard to define [36]. Apaydin et al. reported that the safe zone for the superior gluteal nerve was smaller than previously reported and that a minimally invasive anterolateral approach may particularly compromise braches to the tensor fasciae latae muscle [39]. Posterior, lateral, or anterolateral approaches to the hip should take into account the exit point of superior gluteal nerve and the distribution of its branches [39].

Using the direct anterior approach, no visible damage was observed to the gluteal superior nerve as the incision is not affecting the gluteal nerve at all. Harm to the gluteal nerve can be done by overstretching it using extra force on the retractors during surgery. Lüdemann et al. measured muscle trauma in 25 patients, who underwent minimally invasive hip arthroplasty involving the direct anterior approach preoperatively and after a 6-month follow-up detected an increased fatty degeneration to the tensor fasciae latae [40]. Meneghini et al. reported a muscle damage to the tensor fasciae latae in all cadavers, which underwent total hip arthroplasty involving the direct anterior approach [41]. Grob et al. stated that the tensor fasciae latae surface was mostly damaged in the midsubstance of the muscle after total hip arthroplasty involving the direct anterior approach, which is exactly the area where the superior gluteal nerve enters the tensor fasciae latae [24]. However, damage to the tensor fasciae latae does not automatically imply damage to the nerve branches, but it does endanger the nerve that is very superficial in this area [24]. Terminal nerve branch lesions of the superior gluteal nerve are probably underdiagnosed because they are not always symptomatic and patients still showed excellent clinical and functional result identical to a modified anterolateral approach [24]. Oldenrijk et al. observed in their study that no muscle damage occurred in four out of five cases and no complications occurred regarding the superior gluteal nerve when using the direct anterior approach [42]. In the anterolateral approach, the superior gluteal nerve was dissected in four cases out of five [42]. Controlling soft tissue retracting force may help to prevent compression neuropraxia [43].

Rachbauer et al. reported complications affecting the lateral femoral cutaneous nerve, but confirms the minimization of the risk in damaging the gluteal superior nerve [20]. Oldenrijk et al. also reports lateral cutaneous femoral nerve damages, while using the direct anterior approach [42].

A limitation of the study was that the superior gluteal nerve was not referenced to the direct anterior interval, as adequate reference points were hard to determine. Measurements were taken with a flexible ruler and anatomical landmarks were marked with needles, which lead to some measurement deviations. Considering the relatively high standard deviation, which resulted from the anatomical differences of the cadavers, the measurement deviations due to the measurement setup are from minor importance.

The study shows that the direct anterior approach for total hip arthroplasty saves the superior gluteal nerve, which is, when damaged, one of the major sources of postoperative muscular dysfunction in this area. In agreement with other publications, we were able to demonstrate that branches of the superior gluteal nerve lie within 19–61 mm proximal to the tip of the greater trochanter in the interval between the gluteus medius and tensor fasciae latae [6, 26]. Damage to these branches can occur in an antero—lateral or a direct lateral approach [6, 44].

## Conclusions

Two intervals to access the hip joint to perform total hip arthroplasty are the direct anterior interval and the Watson–Jones interval. In both, total hip arthroplasty can be performed with only minimal muscle damage. No visual damage occurred to the superior gluteal nerve, as the incision is not affecting the gluteal nerve at all. Harm to the gluteal nerve can be done by overstretching it using extra force on the retractors during surgery. Special care should be paid to the area, where the superior gluteal nerve enters the tensor fasciae latae, to reduce the risk in gluteal nerve insufficiency.
